# Prediction of Dengue Outbreaks Based on Disease Surveillance and Meteorological Data

**DOI:** 10.1371/journal.pone.0152688

**Published:** 2016-03-31

**Authors:** Aditya Lia Ramadona, Lutfan Lazuardi, Yien Ling Hii, Åsa Holmner, Hari Kusnanto, Joacim Rocklöv

**Affiliations:** 1 Department of Public Health and Clinical Medicine, Epidemiology and Global Health, Umeå University, Umeå, Sweden; 2 Center for Environmental Studies, Universitas Gadjah Mada, Yogyakarta, Indonesia; 3 Department of Public Health, Faculty of Medicine, Universitas Gadjah Mada, Yogyakarta, Indonesia; 4 Department of Radiation Sciences, Umeå University, Umeå, Sweden; Centro de Pesquisas René Rachou, BRAZIL

## Abstract

Research is needed to create early warnings of dengue outbreaks to inform stakeholders and control the disease. This analysis composes of a comparative set of prediction models including only meteorological variables; only lag variables of disease surveillance; as well as combinations of meteorological and lag disease surveillance variables. Generalized linear regression models were used to fit relationships between the predictor variables and the dengue surveillance data as outcome variable on the basis of data from 2001 to 2010. Data from 2011 to 2013 were used for external validation purposed of prediction accuracy of the model. Model fit were evaluated based on prediction performance in terms of detecting epidemics, and for number of predicted cases according to RMSE and SRMSE, as well as AIC. An optimal combination of meteorology and autoregressive lag terms of dengue counts in the past were identified best in predicting dengue incidence and the occurrence of dengue epidemics. Past data on disease surveillance, as predictor alone, visually gave reasonably accurate results for outbreak periods, but not for non-outbreaks periods. A combination of surveillance and meteorological data including lag patterns up to a few years in the past showed most predictive of dengue incidence and occurrence in Yogyakarta, Indonesia. The external validation showed poorer results than the internal validation, but still showed skill in detecting outbreaks up to two months ahead. Prior studies support the fact that past meteorology and surveillance data can be predictive of dengue. However, to a less extent has prior research shown how the longer-term past disease incidence data, up to years, can play a role in predicting outbreaks in the coming years, possibly indicating cross-immunity status of the population.

## Introduction

The incidence of dengue has grown dramatically around the world in the last few decades [1,2]. Recent research estimates that there are 390 million dengue infections per year and predict that dengue transmission is ubiquitous throughout the tropics, with the highest risk in the Americas and Asia regions [2]. The economic burden of dengue on endemic countries are large and country case studies unveiled rough estimates for 2011 outbreak costs of, for example, 4.5 million US$ in Peru, 6.75 million US$ in Indonesia and 12 million US$ in Vietnam (all in 2012 US$) [3].

The dengue incidence patterns in the Southeast Asia Region are periodic and the situation of an epidemic in various countries varies greatly from time to time and from place to place [4]. Indonesia has been estimated the country with the highest economic burden of dengue in the region [5]. Dengue in Indonesia was first reported in 1968 in Jakarta (DKI Jakarta) and Surabaya (East Java). Incidence of dengue over the past 45 years increased rapidly with the highest incidence rate shifting from young children to older age groups [6]. Generally, the pattern of dengue cases is inversely related to the patterns of mortality rate, but also underlying this aggregated trend heterogeneous temporal and spatial patterns emerge. The case fatality rate (CFR) approximated to 41% when first reported in 1968, but has declined since then and estimated 0.7% in 2013 [6]. In recent time, the dengue geographical transmission pattern in the study area have changed from it being an almost entirely urban disease to becoming much more prevalent in rural areas [7]. However, most of the reported dengue cases are from areas with high population density, such as provinces in Java, Bali and Sumatra [6,8].

Dengue infection has a broad clinical illness spectrum, ranging from asymptomatic, or undifferentiated febrile illness (viral syndrome), dengue fever (DF), dengue hemorrhagic fever (DHF), to dengue shock syndrome (DSS) [9]. Dengue infection may cause complications in the nervous system and other clinical complications, with sequelae or fatal consequences [10]. Clinical management of dengue infection is generally relies on body fluid handling, and proper management save lives [9]. Severe dengue condition only occur as a small part of the overall dengue burden, and it remains difficult to identify patients who have higher risk of sever disease [11].

Exposure to one serotype of the four dengue viruses serotypes (DENV1-4) leads to lifelong immunity to infection of the specific serotype [12], and boost shorter-term cross-protection against other serotypes [13] for approximately 1–3 years [14]. However, vaccines for dengue is developing, today it remains difficult to balancing between neutralizing antibody responses to all four serotypes and remain high efficacy of immunization [11]. Sequential infection of different serotypes of dengue appears to increase the risk for a more severe disease [15].

The primary vector of dengue, Aedes aegypti mosquitoes, adapt well to human environments. It prefers to rest inside the house and to feed during the day [16]. In order for transmission to occur, the female Aedes aegypti mosquito must bite on the infected person during the viraemic phase of the illness [9]. After entering the mosquito through the blood meals, dengue virus requires additional 6–15 days for incubation before it can then be transmitted to another person [17].

Vector and virus dynamics are sensitive to changes in meteorological pattern. For example, Aedes larvae have been found mostly in the rainy season because rainfall contributes to generate vector-breeding sites [18,19]. However, the relationship between rainfall and dengue is non-linear, and heavy rainfall can severely affect vector abundance downwards [19]. Temperature and humidity influence vector biology and vector-virus interactions, through vector longevity, mating, dispersal, feeding behavior and oviposition, as well as a more rapid replication of the virus [20,21]. Associations between rainfall, temperature and humidity to dengue cases, without the intermediate steps of mosquito life-cycle, have been observed in several areas [19,22,23].

The dengue control program in Indonesia aims to halt and prevent the transmission of disease through vector control [8]. Vector control is widely used and the methods to control dengue, but is frequently unsuccessful [24]. One main reason for vector control being less successful is thought related to the poor and reactive management targeting interventions too late in the epidemics. Thus, dengue control, through early warning systems, may show much more effective [8].

An existing early warning for dengue in Yogyakarta has been able to categorize an epidemic year based on disease surveillance and sea surface temperature anomalies data in the preceding months of October and November [25]. However, this system has its limitation as it only tries to estimate the nature of the next year's epidemic activities beginning in March. Meanwhile another study in Yogyakarta shows that there is more than one peak of the monthly number of dengue cases that took places throughout the year [26]. In addition, a study conducted in Singapore provides evidence that dengue can be forecasted with good precision up to 16 weeks ahead by the use of appropriate disease surveillance data and information on the meteorological conditions prior the onset of the outbreak [27]. Therefore, it may be possible to develop an early warning that is able to estimates the nature of the next one or two month's epidemic activities for Yogyakarta.

Therefore, this study set out to study predictiveness of dengue transmission in Yogyakarta Municipality using meteorological and surveillance data. The objectives of this study are: (1) to describe the relationship between predictors on subsequent dengue transmission; and (2) (3) to find and optimal combination of predictors and estimate predictive ability on external data.

## Materials and Methods

### Study Area

Yogyakarta Municipality is one of the five districts and the capital of Yogyakarta Province, Indonesia. It is located about 538 km from Jakarta and 329 km from Surabaya. The two largest cities in Indonesia where dengue was first reported in1968 [8]. Yogyakarta Municipality is geographically located between 110°20’41” to 110°24’14” East Longitude and 07°45’57” to 07°50’25” South Latitude, on the south central of Java Island. The topography is characterized by slope terrain much influenced by Merapi volcano, with the altitude is between 75 to 132 m above sea level. The Yogyakarta Municipality area is 32.5 km^2^, and divided into 14 sub districts and 45 villages [28]. Official reports for Yogyakarta Province in 2013 mention 390,553 persons habituating in the area, and a population density of 12,017 people per km^2^. The population density for the whole Yogyakarta Province is considerably lower and amounts to 1,095 people per km^2^. The annual population growth rate in Yogyakarta Municipality is below 1% during 1990–2010, thus the population was relatively stationary during the study period (S3 File).

**Fig 1:**
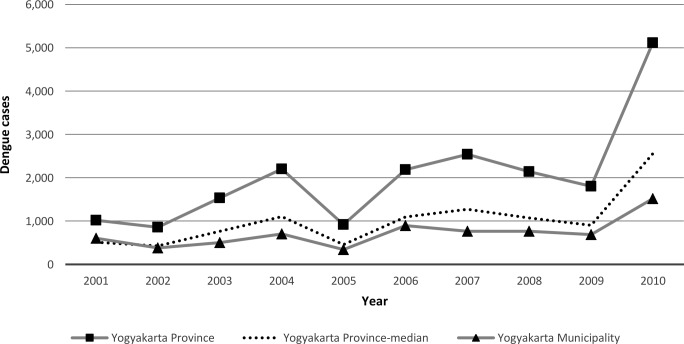
The Number of Dengue Cases in Yogyakarta Province, 2001–2010.

Based on Yogyakarta Province Health Profile in 2011, DHF Incidence Rate per 100,000 Population for Yogyakarta Municipality is estimated to 105, compared to the DHF Incidence Rate for Yogyakarta Province, which is estimated to 29. Yogyakarta Municipality contributes most to dengue among the five districts in the Yogyakarta Province. For the years 2001, 2002, 2005, 2006, and 2009, the number of dengue patients in the Yogyakarta Municipality contributes to around 50% of all dengue patients in the Yogyakarta Province (**Fig 1**). The municipality has a total area of around 1% of the total area of Yogyakarta Province, and almost all villages in Yogyakarta Municipality report dengue cases [26].

### Data

In this study, we have used aggregated monthly dengue cases (dengue fever + dengue hemorrhagic fever + dengue shock syndrome) at municipality level from the Dengue Surveillance Report, Yogyakarta Municipality District Health Office, for the period 2001–2013. Meteorological data from 2001–2013 was obtained from the Indonesian Agency for Meteorology, Climatology and Geophysics (BMKG). Records of cumulative monthly rainfall (mm), mean monthly temperature readings (°C), and mean monthly relative humidity (percentage) were obtained. The meteorological data was merged to the aggregated total number of confirmed dengue cases per month.

A model was developed and validated by dividing the data file into two datasets: the data from January 2001 to December 2010 was used to train a model, and data from January 2011 to December 2013 was used for testing and validate the fitted model. There were only one missing observation for temperature, rainfall and relative humidity in the training dataset, meanwhile there were eight missing observation for temperature, two missing observation for rainfall and seven missing observation for relative humidity in the testing dataset. Since the amount of missing data is very small relatively to the size of the training dataset we did not impute missing data. In a sensitivity analysis, in the supplementary material, we show that imputation does not contribute to better model fit (S2 File).

### Statistical Analysis

The analyses focus on studying the meteorological variables relationship to the dengue time series, as well as the self-inherit predictiveness within the dengue time series itself. The analyses compose of a set of prediction models and compare predictive ability to the disease count, to detect and predict the temporal patterns of dengue and dengue outbreaks. According to previous findings there is evidence for a relationship between weather and dengue transmission with up to a 16–20 weeks delay between the variability in the weather factors and corresponding influences on the dengue cases [27]. We therefore decided a priori, for weather, to use lag times with up to 4 months delay in the analysis, thus, including lag 0–3 of the meteorological variables.

Using standard correlation analysis, we found the rainfall variable to be highly correlated to the relative humidity variable. As the high correlation may give rise to singularity problems when fitting a statistical model, we addressed this by subtracting the co-variation of temperature and rainfall in the relative humidity series using a generalized linear model with a Gaussian link function and allowing the temperature and rainfall to co-vary to humidity using a non-linear natural cubic spline function with 3 degrees of freedom per variable according to:

$$RH_{t}∼\;\alpha \;+\;ns\;\left({TMP}_{t},\;df=3\right)\;+\;ns\;\left({PRE}_{t},\;df=3\right)$$ (1)

Where *t* corresponds to time in months taking the values 1 to 120 from the start of the study January in 2001 to the end of the study period December in 2010. *RH*_*t*_ is the measurement of relative humidity on month *t*, *α* corresponds to the intercept, ns() denotes a natural cubic spline, *TMP*_*t*_ and *PRE*_*t*_ correspond to the readings of temperature and rainfall in month *t*, and *df* defines the degrees of freedom (and determines the number of knots) for the natural cubic spline function.

In the subsequent analyses, we used the residuals from above model as to represent relative humidity adjusted for temperature and rainfall co-variation. The residuals can be expressed as: *RHres*_*t*_ = *RH*_*t*_ − *fitted*(*RH*_*t*_) from the model **(**1**)** above. The residuals of relative humidity are, thus, independent and are not correlated to the temperature and rainfall. The reasoning for taking this approach was that we expected most of the variability in relative humidity over the months to be, in fact, intermediate to rainfall levels, as in combination with temperature levels it largely determines evaporation. As a consequence, we managed to overcome potential collinearity.

The initial analyses of meteorological variables and their association to the disease patterns over time were established using all lag of month 0–3 of temperature, rainfall and adjusted humidity in the model at the same time. This approach was taken as to disentangle the various lag contributions influences of the same meteorological variable in the results. The patterns were studied in relation to disease counts assuming a quasi-Poisson distribution and a log link function of the dengue count data allowing for over-dispersion. In supplementary information (S1 File) we illustrate the small difference in model fit when making a Negative Binomial distribution assumption versus a quasi-Poisson distribution assumption. The generalized linear regression models fitted allowed non-linear relationships between the weather variables and the dengue outcome variable by using natural cubic spline functions with 3 degrees of freedom per variable. The regression model can be expressed as:

$$\text{log}(D_{0,t})∼\alpha +\sum_{l=0}^{3}ns(TMP_{lt},df=3)+\sum_{l=0}^{3}ns(PRE_{lt},df=3)+\sum_{l=0}^{3}ns(RHres_{lt},df=3)$$ (2)

Where log() denotes the natural logarithm, *t* corresponds to time in months, *D* correspond to the dengue count, the first index, *l*, denotes the lag variables, ns() denotes a natural cubic spline, and *TMP*_*t*_, *PRE*_*t*_ and *RHres*_*t*_ correspond to the readings of temperature, rainfall and relative humidity adjusted for rainfall and temperature variation, and *df* denote the degrees of freedom for the natural cubic spline function.

Sensitivity analyses were performed on these analyses, and included adding a time trend variable to account for between year variability in the dengue series not related to the other factors included. The time trend function was modelled using a natural cubic spline function of 4 degrees of freedom. Graphical interpretations of the relationship between the variables studied were generated for the natural cubic spline functions.

#### Surveillance data as predictor

In order to build models that could use recent, and historic disease surveillance in the predictions, we assed first how lags of past dengue counts affected the dengue transmission, such relationships can be seen as a variant of autoregressive (AR) patterns in time series. The first part of this analysis aimed at estimating how the most recent transmission influencing the dengue counts using the same lags as for the meteorological variables, 1–3. However, for obvious reasons excluding lag 0 as it is the in fact the outcome variable. This model-resembled model (2) above but interchanging the meteorological variables to the dengue variable and removing lag 0 from the predictor side of the equation. The rationale for assessing these relationships was to capture patterns in the disease transmission influenced by non-weather factors, such as those driven by population level immunity and cross-immunity, which increase after large outbreaks.

Prior studies, on a finer spatial scale, have shown that the recurrence of dengue heterotypic cases in a spatial location may be reduced up to years time [29]. In a similar way, now on a coarser spatial scale, our interpretation of the reverse relationship between dengue up to 24 months back and current transmission risk may indicate a similar mechanism on a population level through cross-immunity. In general, this pattern may also explain and mediate some of the complex inter-annual returning cyclic patterns of dengue observed in endemic settings. These analyses set out to study if also longer lag time up to four years prior explained variability in the disease transmission dynamics. The underlying reasoning for this analysis was to assess and estimate if the disease transmission risk would increase if the retrospective transmission (1–48 months back) had been lower or higher. We hypothesized that this would function as a proxy for susceptibility of the populations to infections. To assess this we used distributed non-linear lag models as provided in the DLNM package of the statistical software R. The methods and implementation of DLNM have been described before [30]. The variable component of the DLNM function used natural cubic spline function, while the lag relationships where estimated using polynomials. The model established was a generalized linear regression models with a Poisson log link function that allowed for over-dispersion. The model is described below:

$$\text{log}(D_{0,t})∼\alpha +\sum_{l=1}^{47}DLNM(D_{l,t},df_{\text{var}iable}=4,df_{lag}=4)$$ (3)

Where log() denotes the natural logarithm, *t* corresponds to time in months, *D* correspond to the dengue count, the first index, *l*, denotes the lag of the dengue count variable, DLNM() denotes a distributed non-linear lag model, and *df* denote the degrees of freedom for the *D* and the lag function.

#### Incidence predictions

We predicted the disease patterns of dengue transmission in Yogyakarta Municipality using the meteorological variables, and the surveillance data on its own, and together. In the prediction models, only lags on a time distance longer or equal to 2 was included to provide at least a one month window between observations of the disease and the meteorology to the prediction of the dengue case counts and potential outbreaks. We established models, and predicted from models, including only explanatory variables of: meteorology at different lags; dengue count at different lags; and combinations of meteorological and dengue count variables at different lags. Thereafter, models were reduced to only include variables that contributed sufficiently to explain and predict the disease according the Akaike Information Criterion (AIC). A difference in AIC larger than 10 was considered sufficient a difference between models to benefit the more complex model among pairs compared. We refer to the models as:

1. Month of the year model
2. Meteorology model (may include lag 2,3)
3. Surveillance model 1 (may include lag 2,3)
4. Surveillance model 2 (may include lag 2–48)
5. Model including an optimal combination of models B-D

A necessary time latency of at least 2 months lag was evaluated to allow up to 2 months lead time in controlling the disease at forecast of epidemics. Graphical time series of the prediction contrasted to the observed disease counts were generated for all prediction models. Models were evaluated based on the adjusted R-squared initially, and later onward by prediction performance according to root mean square error (RMSE) and standardized root mean square error (SRMSE) for continuous predictions.

#### Outbreak predictions and validation

For the final prediction model derived, we established predictions and calculated the accuracy of the prediction according to a binary classification of months as outbreaks (or epidemic), or non-outbreaks. For this purpose, a constant epidemic threshold was applied defining outbreaks/epidemics as months with more than 60 confirmed cases. This assumption was relaxed, and also other established definitions of outbreaks were used (S4 File). Prediction accuracy according to sensitivity, specificity, and positive and negative predictive values were calculated. In the next step, the final model is externally validated by predicting dengue cases for the period January 2011 to December 2013, and comparing with observations collected for this time period.

All analyses were performed in R [31] using the mgcv package [32]. Datasets and code needed to reproduce the results presented here are available on github at https://github.com/alramadona/yews4denv.

## Results

**Fig 2:**
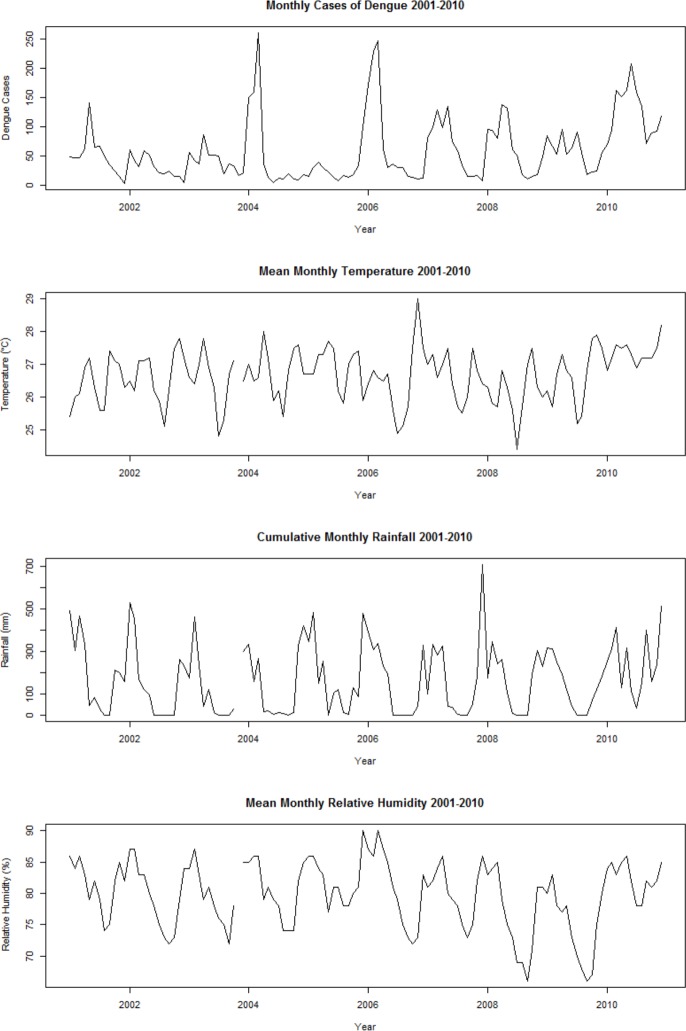
Time Series Graphs of Surveillance and Meteorological Data.

The total count of dengue cases in the 120 months study period, from January 2001 to December 2010, was 7,171 with a monthly average of 60 confirmed cases, a maximum of 261 and a minimum of 3. The dengue incidence increased gradually from December to March in the following year, and then decreased until the start of the rains the next coming year (**Fig 2**). This study indicates a surge in the number of cases that increased about two times over the study period (**Fig 1**), from 343 and 688 cases in 2005 and 2009, and then surged to 894 and 1,517 cases in 2006 and 2010.

Overall, the monthly mean temperatures during study period ranged from 24 to 29 ^o^C. Yogyakarta experienced the lowest monthly mean temperature, 24 ^o^C, in July 2008 and the highest monthly mean temperature, 29 ^o^C, in November 2006. The highest amount of monthly cumulative rainfall was recorded to 709 mm in December 2007, with an average of 166 mm. Meanwhile, minimum and maximum value for the monthly mean relative humidity was 66% and 90% respectively.

Based on the average of monthly cumulative rainfall and the average of monthly cases we observed a phase difference of three months. The average of monthly cumulative rainfall was the lowest occurred in August (17 mm), while the average of monthly cases was the lowest occurred in November (25 cases), and the average of monthly cumulative rainfall was the highest occurred in December (357 mm), while the average of monthly cases was the highest occurred in March (109 cases).

**Fig 3:**
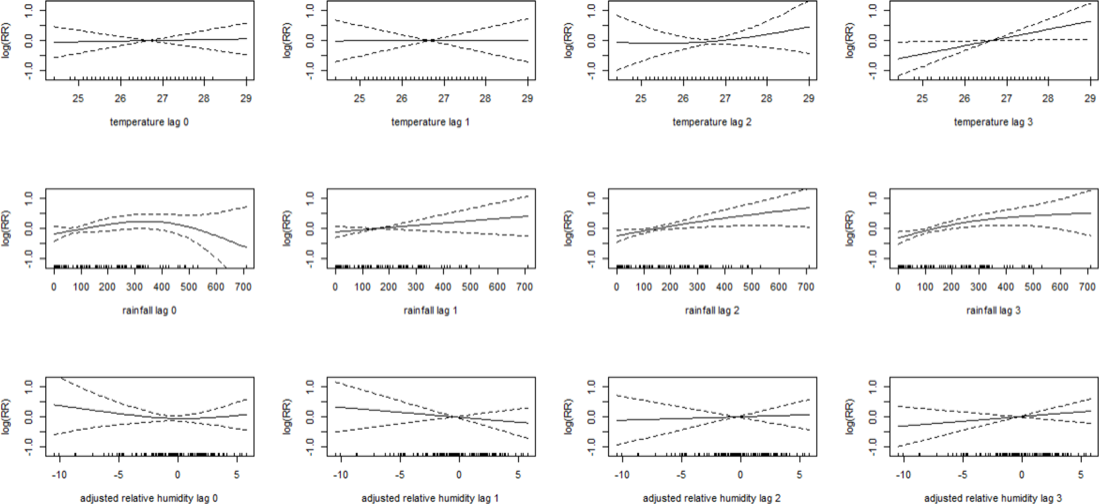
Association between Meteorological Variables and Dengue over Lag 0–3. Solid lines represent relative risks of dengue cases and dotted lines depict the upper and lower limits of 95% confidence intervals.

Temperatures showed low association with dengue cases in lag 0, 1 and 2, but in lag 3 the relative risk increase linearly when temperature increase. Rainfall in lag 0 associate to a slightly increasing dengue transmission with lower levels, but indicate a strong drop when the monthly rainfall is more than 300 mm. Lag 1, 2 and 3 of rainfall indicate a linear increase of the relative risk with more rainfall. But also here, the increasing relative risk of dengue transmission vanishes when the rainfall is very high. Adjusted relative humidity did not show a consistent pattern, but indicated linear increase in lag 2 and 3, and linear decrease in lag 1 with increasing humidity levels (**Fig 3**).

For prediction models, when only lags longer or equal to 2 were included, the cross-correlation (S1 Fig) indicate that the highest positive association between dengue incidence and temperature were found at lag 3 (r, 0.35) and rainfall at lag 2 (r, 0.62). The adjusted relative humidity at lag 2 and lag 3 indicated a small and negative correlation with dengue incidence (r, -0.01 and -0.12). Meanwhile, the analyses using all lag of month 0–3 of temperature, rainfall and adjusted humidity in the model at the same time (S1 Table) showed that statistical effects appear at temperature in lag 3 (p-value, 0.03), rainfall in lag 2 (p-value, 0.02) and rainfall in lag 3 (p-value, 0.01). On these bases, and after trying different combination to identify the optimal model according R-squared, RMSE, SRMSE, and AIC, showed consistent results that the optimal meteorological predictor variables for the study area is temperature at lag 3, rainfall at lag 2 and rainfall at lag 3.

**Fig 4:**
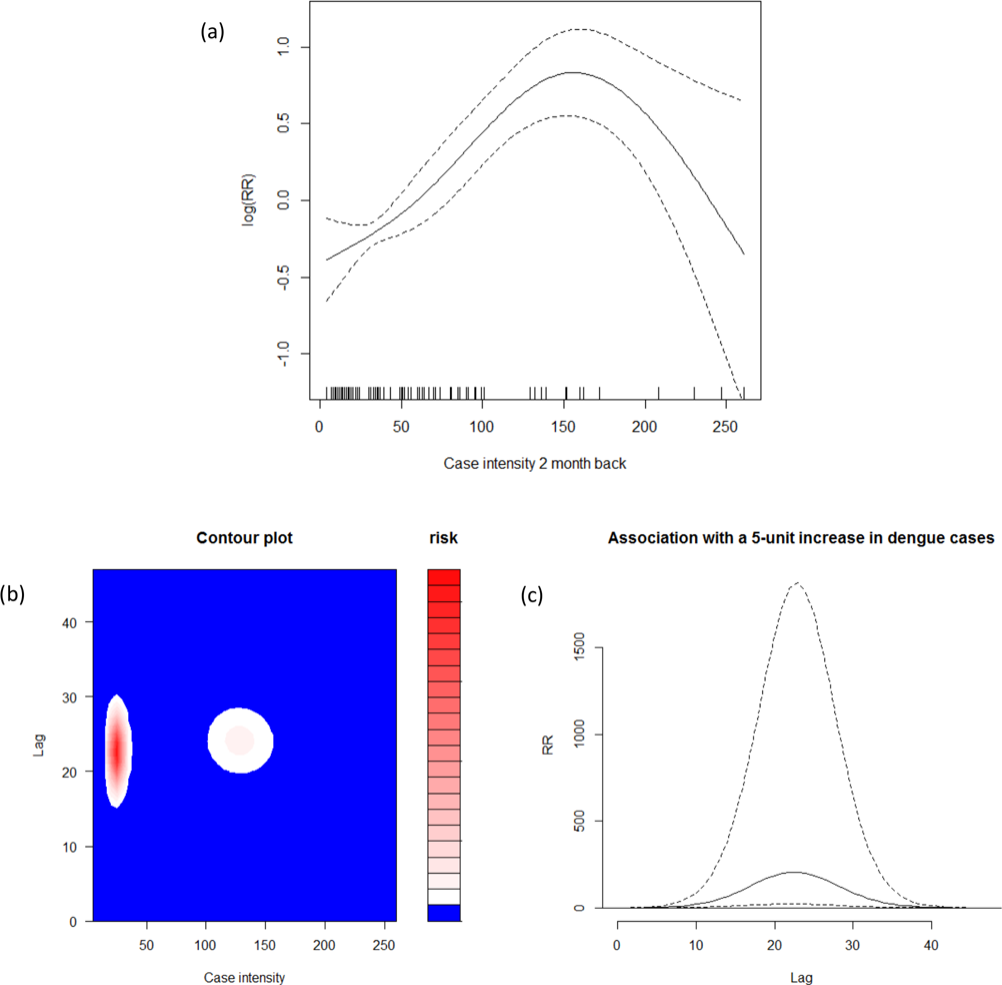
Relationship between Autoregressive Lags and Dengue Counts. Upper panel shows (a) relative risks of dengue cases as functions of dengue surveillance at 2-month lag times. Lower panel shows (b) the relation between case intensity and dengue risk categories at all lag months; and (c) the risk in each future month following an increase of 5 dengue cases in a specific month.

The highest correlations between dengue incidence and lagged dengue incidence, with lag terms within a few months and when only lags longer or equal to 2 were included, were found at lag 2 (r, 0.47). Dengue cases at lag 2 months back show associations to increase dengue transmission with lower values, but to decreasing when the dengue cases counts is more than 150 (Fig 4A). Meanwhile, for lag within longer-term months, the non-linear distributed lag models showed a peak around lag 24 (Fig 4B) suggesting a negative feedback cyclic pattern with lower relative risks of transmission up to two years following a large outbreak in around lag 24. An increase of the dengue cases counts in a specific month will be increasing the dengue risk in each following month with a peak at approximately in the next 24 months (Fig 4C). Based on these, the optimal variables for the prediction models included dengue count at lag 2 and at lag 24.

### Predictive Performance of Models

**Fig 5:**
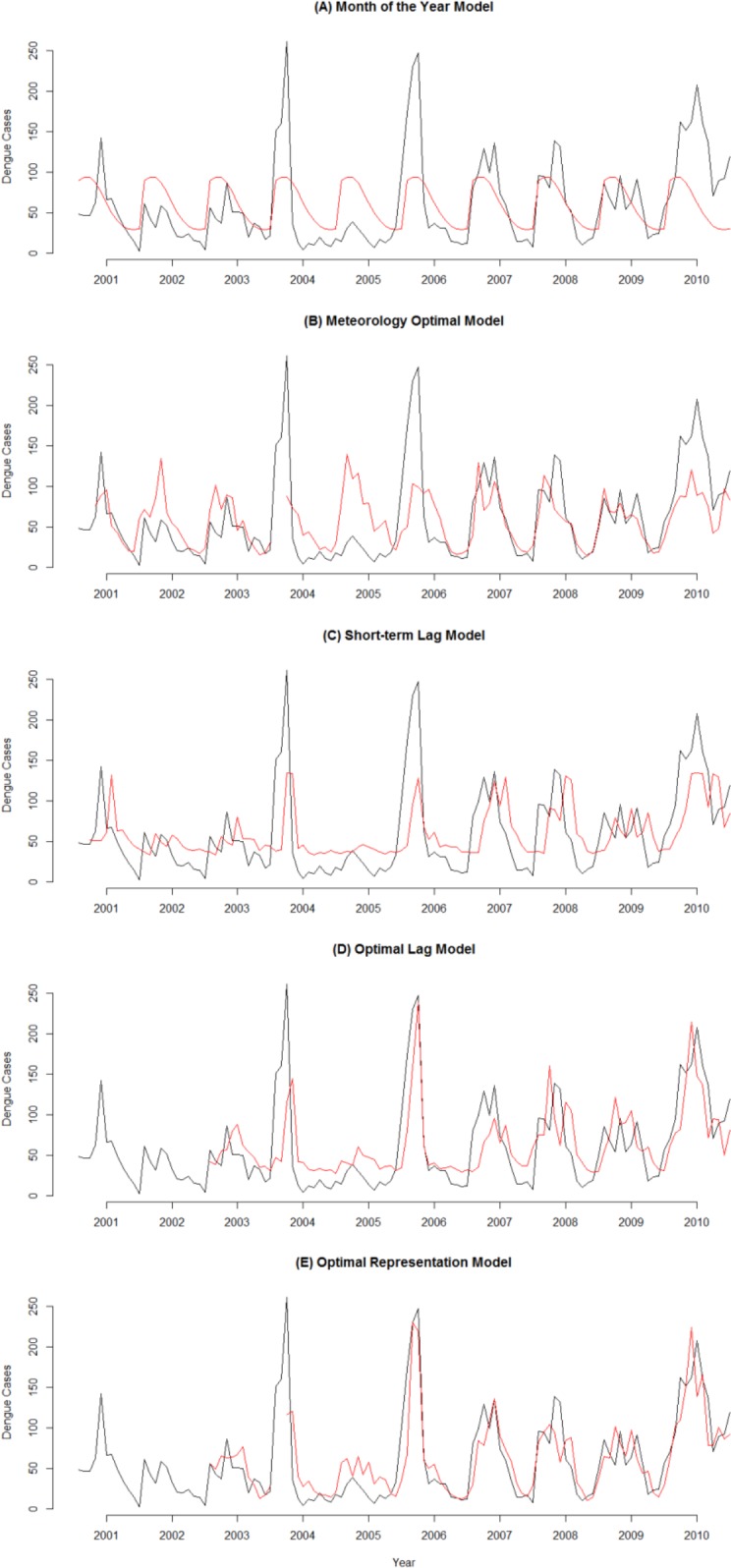
Monthly Observed and Predicted Dengue Cases from 2001–2010. Black line represents observed dengue cases and red line represents predicted cases. The vertical axis shows dengue cases and the horizontal axis denotes time in month from January 2001 to December 2010.

The simplest model (Model A) using the months of the year showed poorest performance when compared with another model. Meanwhile the model using the optimal combination of meteorological factors (B) only (temperature at lag 3, rainfall at lag 2 and rainfall at lag 3) showed a relatively good fit and reasonable predictive ability. The model included a dengue count time series at lag 2 (C) showed, however, poor predictions, but the optimal autoregressive model including dengue at lag 2 and lag 24 (D) showed a rather good predictive performance that was comparable to the meteorological based model (**Fig 5**). The predictive ability as evaluated by RMSE and SMRSE, as well as the values of AIC for the models A-E consistently shows that model (E) combining model (B) and (D) is the best-predictive model (**Table 1**). The final model (E) included combinations of the meteorology and the autoregressive lag terms of dengue counts in the past according to:

$$\text{log}(D_{0,t})∼\alpha +\sum_{l=3}^{3}ns(TMP_{lt},df=3)+\sum_{l=2}^{3}ns(PRE_{lt},df=3)+\sum_{l=2}^{2}ns(D_{lt},df=3)+\sum_{l=24}^{24}ns(D_{lt},df=3)$$ (4)

**Table 1:** Predictive Performance Statistics

Model	RMSE	SRMSE	R-sq.(adj)	AIC	∆ AIC
(A) Month of the Year	46.633	0.580	0.229	4884.18	0.00
(B) Meteorology Optimal	44.540	0.553	0.282	3867.23	-1016.96
(C) Surveillance: Short-term Lag	45.046	0.560	0.294	4362.54	-521.65
(D) Surveillance: Optimal Lag	41.846	0.520	0.443	3228.67	-1655.52
(E) Optimal Representation B-D	32.448	0.403	0.636	2311.60	-2572.58

∆ AIC = change in Akaike Information Criterion compared to the simplest model (A)

**Fig 6:**
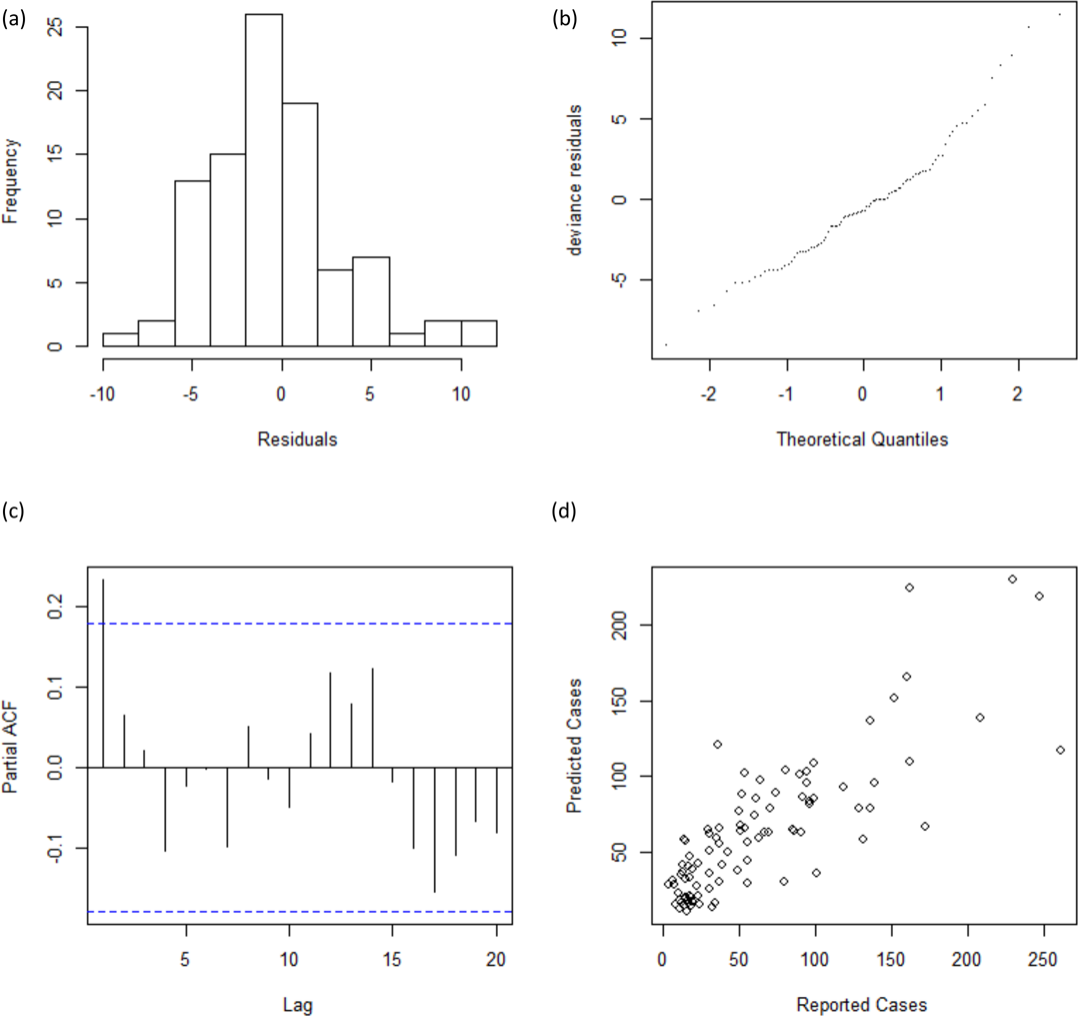
Residual Diagnosis. Upper panel shows (a) the residual histograms; and (b) the Q-Q plot for deviance residuals. Lower panel shows (c) the partial ACF plot; and (d) the relationship between reported and predicted cases.

After fitting the model, we infer residuals and check them for normality and residual autocorrelation. Residual histograms exhibited a single modal and almost symmetrical pattern. In addition, the Q-Q plot for deviance residuals presented a reasonably straight line. Thus, suggesting approximate normal distribution of residuals. Meanwhile graphical examination of Partial ACF plot shows insignificant autocorrelation in the residuals, and the plot of reported and predicted cases indicate linear relation (Fig 6).

**Fig 7:**
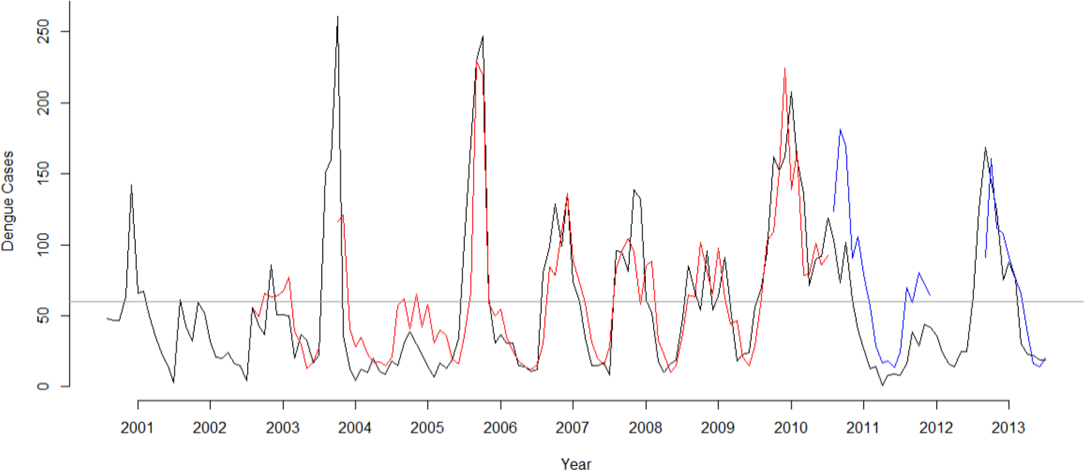
Predicted Dengue Cases Versus Reported Dengue Cases in 2001–2013. Monthly predicted dengue cases compared with reported cases during January 2001 to December 2013. Black line represents observed dengue cases, grey line represents the epidemic threshold, red line represents predicted cases using training dataset, and blue line represents predicted cases using the external validation dataset not used for model fitting.

The estimated relationships in the final model (E) were used for the predictions. Using the (E) model, predictions showed a relative good discriminating ability to separate transmission months above and below 60 cases, which is as follows: 47 prediction was correctly negative, 32 correctly positive, 4 incorrectly negative, and 11 incorrectly positive (Fig 7). The total error rate of the prediction was 16%. Meanwhile, the sensitivity of detecting the outbreaks was estimated to 88.9% and the specificity to 81.0%. In addition, the positive and negative predictive value respectively was estimated to 74.4% and 92.2%.

During the forecast for 2011–2013, the optimal model forecasted cases versus actual clinical reported dengue cases gave 0.6 of prediction error. Meanwhile, the sensitivity of detecting the outbreaks was estimated to 100% and the specificity to 61%. In addition, the positive and negative predictive value respectively was estimated to 59% and 100%. Missing meteorological data caused absence of predictions for a period of 8 months between June 2012 to January 2013. Thus, predictions could only be made for 28 months out of 36 months (78%). Our model forecasted all the cases above the epidemic threshold, with 7 false positive case at May-June 2011, January 2012, March-May 2012, and August 2013 (Fig **7**).

## Discussion

In this study, the dengue transmission pattern was predicted using disease surveillance and meteorological data. A best model for dengue prediction was identified by combining meteorology and surveillance data as predictor variables. The results support that temperature and rainfall are associated with dengue incidence and epidemic transmission, as well as are short-term lags of disease counts, and longer-term the retrospective disease transmission. Meanwhile, relative humidity not contributed sufficiently to explain and predict the disease.

The relationship between weather and subsequent dengue transmission in this current study is consistent with the evidence of previous research that shown a relationship between weather and dengue transmission with up to four months delay [27], and a study showing that the risk of re-current outbreaks in the same location decreased for a few years when after an outbreak [29,33]. The explanation for this may be both related to the fact that the immunity, or herd immunity, of the population increase after an outbreak subsequently the transmission may cease for some time. The time period in this study of around 2 years may suggest that the risk of outbreaks decrease up to 2 years because of reasons of cross-protection to other serological dengue strains after infection with one [14]. The short-term 2-lag relation shows that recent transmission influences the rate of dengue incidence, whereas longer-term 24-lag relation indicates a cyclic inter-annual periodic pattern.

However, beside intrinsic regulation related to host-virus interactions, principally mediated by serotype-specific immunity, there might be extrinsic drivers such as changes in weather patterns [34] that contribute to a cyclic inter-annual pattern. During study period, there have been three moderate-to-strong El Niño events in 2002–3, 2006–7, and 2009–10 [35]; and it could be have a role to a surge in the number of cases over the study period in 2005–6 and 2009–10 (Fig 1). The impacts of El Niño have been associated with hotter-than-normal conditions in Indonesia [35], while the temperature can affect vector longevity, mating, dispersal, feeding behavior and oviposition, as well as a more rapid replication of the virus [20,21]. Therefore, dengue vector has a shorter time to reproduce and become adult, as well as have a greater probability of becoming infected and infecting another host. However, the El Niño events normally occur with slightly more delayed periodicity.

Our study shows that temperature with three-month lead-time and rainfall with two and three-month lead-time is the best predictors for dengue transmission patterns in Yogyakarta Municipality. The time delay between temperature and rainfall with corresponding influences on the dengue cases have shown in others province in Indonesia [36] and neighbor countries [27,37] as well. This time delays are likely to represent biological processes in the vector life cycle [19,20]. Relative humidity is not found to be a strong predictor in the models, and this reinforce previous studies that have shown relative humidity is not a significant variable in the spatial or temporal distribution of dengue in Indonesia [36]. However, relative humidity appears to be somewhat influential at lag 0 for the dengue incidence in the study area.

Our study has shown that combinations of the meteorology and the autoregressive lag terms of dengue counts in the past (Fig 5E) are able to predict dengue incidence quite accurately, either as transmission numbers or as outbreaks or non-outbreaks periods. Meanwhile, the surveillance models (C-D) visually give reasonably accurate results for outbreaks period, it does, however, not work well for non-outbreaks period (**Fig 5**C and **5**D); interestingly, the meteorology model (B) show the opposite pattern (**Fig 5**B). Combining of both model results in a best-predictive model for dengue outbreaks based on disease surveillance and meteorological data.

Our model forecasted dengue cases up to 2 month ahead and showed a consistent ability to separate months with epidemic and non-epidemic transmission in the training data, as well as in the testing data. The model predicted 10 out of the 10 epidemic months in 2011–2013 correctly. Performance prediction of the model seems very good for predicting dengue incidence in 2013 (SRMSE, 0.3), but less in 2011 (SRMSE, 0.9), and overall suggested that the model forecast cases with sufficient sensitivity for detecting outbreaks for 2011–2013 (SRMSE, 0.6). There are 7 false alarms during 2011–2013. However, most of them occurred in the peak season of dengue incidence. Based on surveillance data from 2001 to 2010, the average monthly incidence of dengue between January-June were more than 60 cases, and between July to December is less than 60 cases.

A number of limitations are apparent for this study. First, our predictive model with disease surveillance and meteorological data as predictors could explain only 64 per cent of the variation in the occurrence of dengue cases. The remaining 36 per cent unexplained variation could be due to the influence of other factors. Second, this study did not perform direct analysis of laboratory surveillance reports, but instead simply used monthly dengue aggregate data. However, the monthly dengue aggregate data was based on dengue surveillance of Yogyakarta Municipality District Health Office that constitutes laboratory confirmed dengue cases only. Third, in the study areas, underreporting could occur with asymptomatic infections and dengue cases that might have self-treatment, or seek care at private clinics [6]. However, dengue is one of the infectious diseases that can cause outbreaks in accordance with National Act No. 4/1984 on Epidemic Diseases, as well as the Minister of Health Regulation No. 560/1989. Therefore, if the physician or health workers discovered or suspected cases of dengue fever, they are required to report to the patient’s local health center in less than 24 hours. Fourth, there is some missing data in the dataset, especially in the validation dataset. However, we believe that imputation does not compensate missing values (S2 File).

Previous studies has shown that the implementation of Dengue Control Programs has not given satisfactory result due to, among others, dengue surveillance program implementation in Indonesia which are mostly passive [6], the difference in communication intensity between the authorities responsible for the Dengue Control Programs, and the different levels of public knowledge about dengue in the community [38]. In addition, dengue vector, Aedes aegypti, have been resistant to some insecticides from the group of organophosphates, and pyrethroids carbamic [39]. Therefore, despite some of the limitations mentioned above, early warning based on this model might be used as a part of the advocacy process for develop a cross-sectorial networking in the surveillance and control of dengue. The models allowed some recognition, although not perfect, of outbreak periods at an early stage with 2 months lead-time. Thus, development of early warning system could benefit from these predictions since one main reason for vector control being less successful is related to the poor and reactive management targeting interventions too late in the epidemics [8]. It may target high-risk periods, for when, health education and public health interventions can effectively prepare communities and potentially curb the epidemic.

In further practice, observed weather could be replaced by weather forecasts, which might extend the lead-time beyond that offered by using lagged observations. Further studies also might extend the model for smaller spatial scales (for example village level) to characterize the relationship between both meteorological and non-meteorological factors and dengue risk, and include more complex feedback in the model as related to temporal and spatial covariance patters. In addition, further research is also needed to explain the intrinsic regulation and extrinsic drivers that might contribute to a cyclic inter-annual pattern.

## Supporting Information

S1 Fig Cross-correlation between Outcome and Predictors. (PDF)

S1 File Outcome Distibution. (PDF)

S2 File Imputation Simulation. (PDF)

S3 File Population Offset. (PDF)

S4 File Comparison of Prediction Accuracy of Several Outbreak Criteria. (PDF)

S1 Table Initial Analyses Summary. (PDF)
