# Supplementary figures and images for: Prediction of Dengue Outbreaks Based on Disease Surveillance and Meteorological Data
Source: PLoS One. 2016 Mar 31;11(3):e0152688. doi: 10.1371/journal.pone.0152688 (PMC4816319; doi:10.1371/journal.pone.0152688)

# Supporting Information

**S1 Fig.** Cross-correlation between Outcome and Predictors

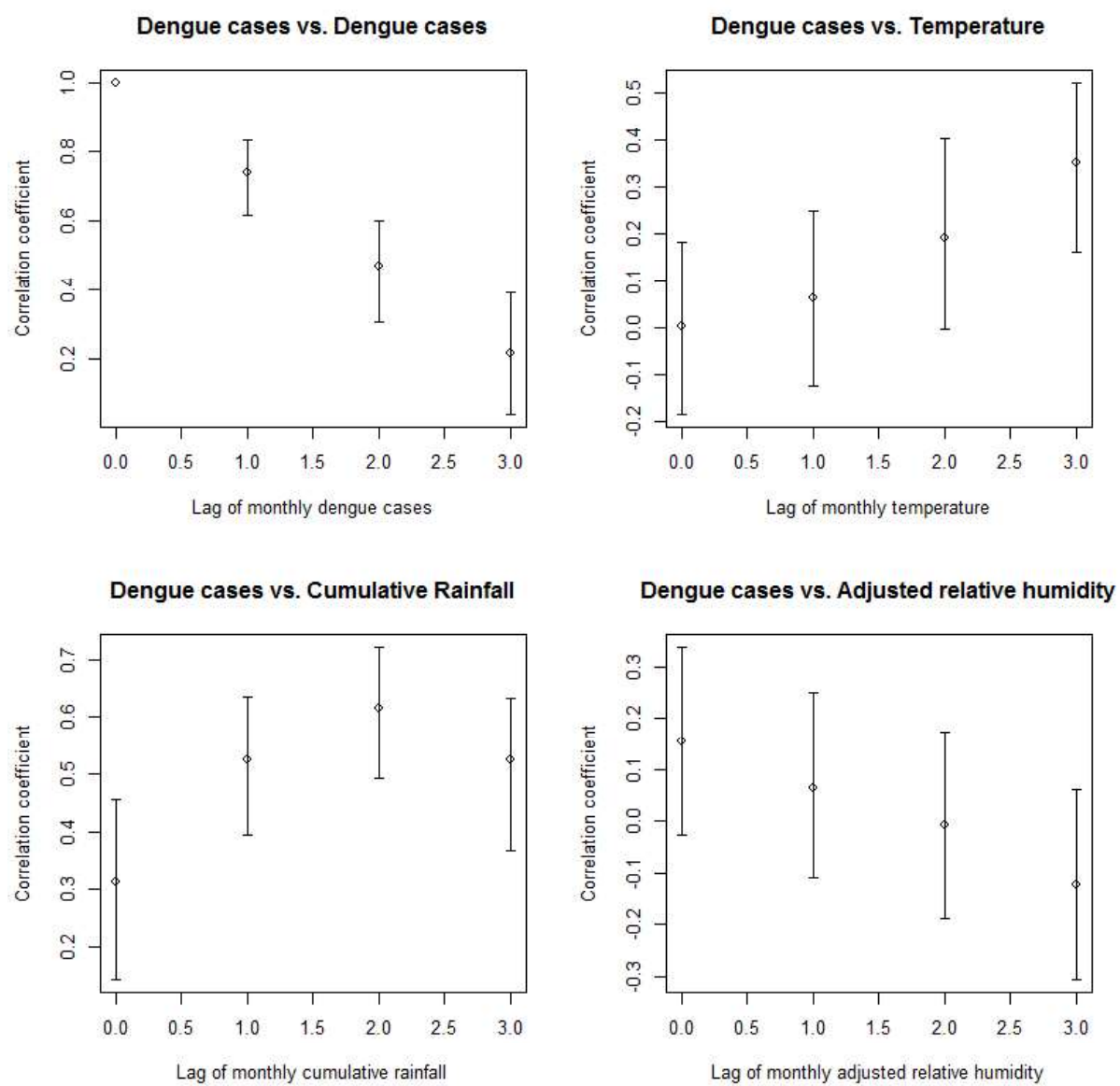

Supplement: S1 Fig — (PDF) [file pone.0152688.s001.pdf]
