# Supplementary material for: Prediction of Dengue Outbreaks Based on Disease Surveillance and Meteorological Data
Source: PLoS One. 2016 Mar 31;11(3):e0152688. doi: 10.1371/journal.pone.0152688 (PMC4816319; doi:10.1371/journal.pone.0152688)
Supplement: S1 File — (PDF) [file pone.0152688.s002.pdf]

S1 File. Outcome Distribution

Diagnostic plot of the variance to mean relationship for dengue dataset, suggests the quasi-Poisson is a better fit to the overall variance-mean relationship.

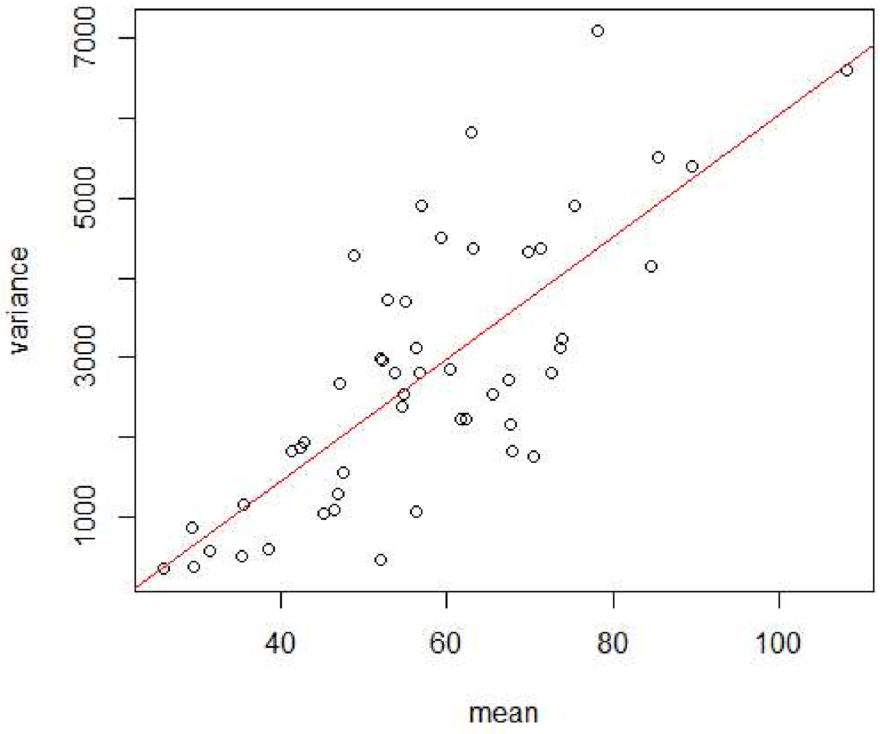

S1 File_Fig 1. Estimated Variance-to-Mean Relationship for Dengue Cases Dataset

The quasi-Poisson regression is providing a little bit better fits in the study.

S1 File_Table 1. Predictive Performance Statistics, quasi-Poisson (QP) vs. Negative Binomial (NB)

**Model**	**RMSE**		**SRMSE**		**R-sq.(adj)**	
	**QP**	**NB**	**QP**	**NB**	**QP**	**NB**
Meteorology Optimal	44.540	44.726	0.553	0.556	0.282	0.275
Surveillance: Short-term Lag	45.046	45.151	0.560	0.561	0.294	0.291
Surveillance: Optimal Lag	41.846	42.347	0.520	0.526	0.443	0.430
Optimal Representation	32.448	33.290	0.403	0.414	0.636	0.620

QP: quasi-Poisson NB: negative-binomial
