# Supplementary material for: Prediction of Dengue Outbreaks Based on Disease Surveillance and Meteorological Data
Source: PLoS One. 2016 Mar 31;11(3):e0152688. doi: 10.1371/journal.pone.0152688 (PMC4816319; doi:10.1371/journal.pone.0152688)
Supplement: S2 File — (PDF) [file pone.0152688.s003.pdf]

## **S2 File.** Imputation Simulation

6% temperature observations were missing, 5% humidity observations were missing, 2% rain observations were missing. There were no missing observations in the aggregated dengue count data.

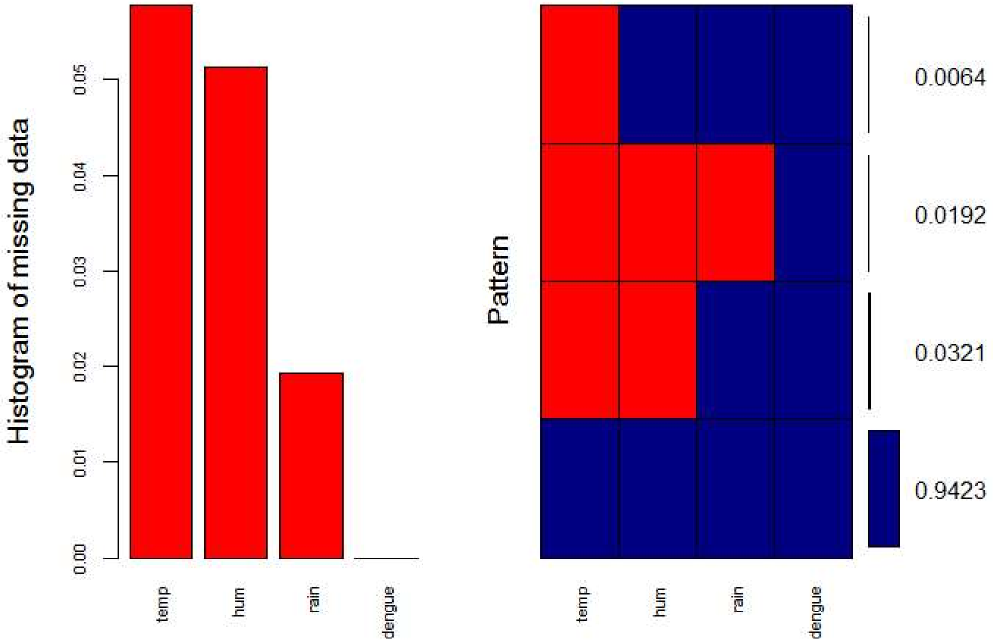


**S2 File_Fig 1.** Dataset Summary without Imputation

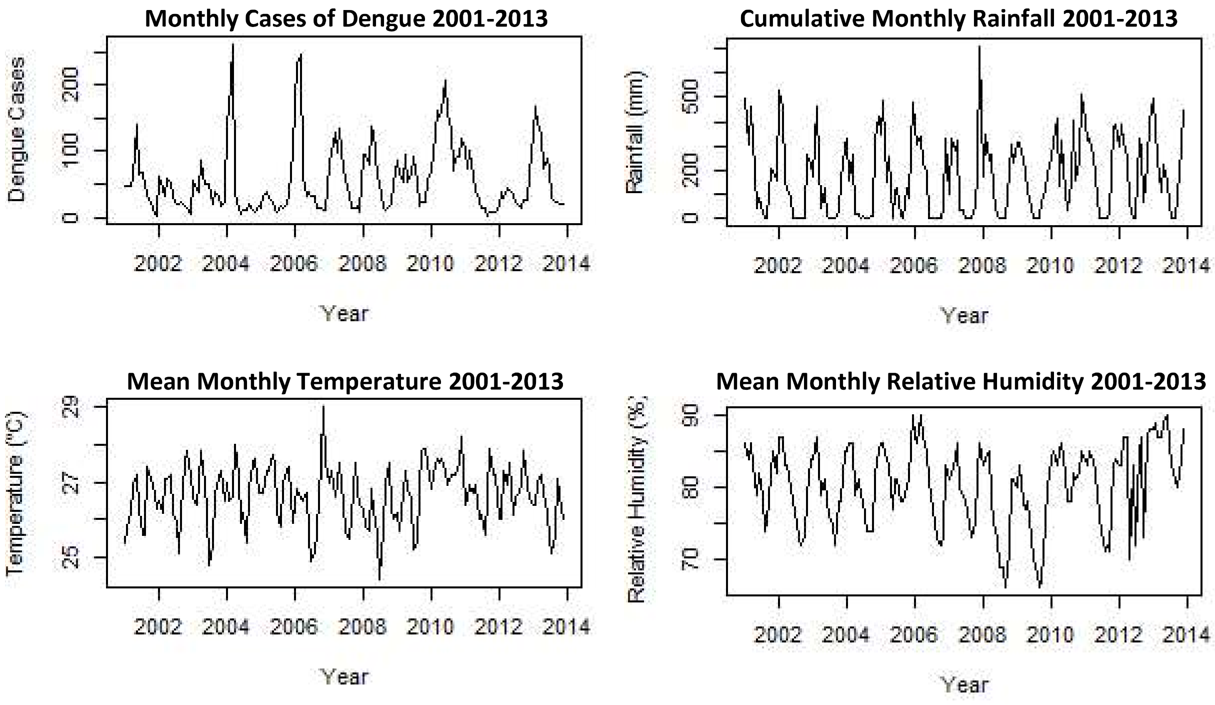


**S2 File_Fig 2.** Dataset Imputation by Predictive Mean Matching

**S2 File_Table 1.** Predictive Performance Statistics, without and with imputation

| Model | Dataset | | | Dataset + imputation | | |
|---|---|---|---|---|---|---|
| | R-sq.(adj) | RMSE | SRMSE | R-sq.(adj) | RMSE | SRMSE |
| Meteorology Optimal | 0.28 | 44.54 | 0.55 | 0.28 | 45.42 | 0.56 |
| Surveillance: Short-term Lag | 0.29 | 45.05 | 0.56 | 0.29 | 45.05 | 0.56 |
| Surveillance: Optimal Lag | 0.44 | 41.85 | 0.52 | 0.44 | 41.85 | 0.52 |
| Optimal Representation | 0.64 | 32.45 | 0.40 | 0.58 | 35.77 | 0.44 |

**S2 File_Table 2.** Model Evaluation, without and with imputation

| Optimal Representation Model | R-sq.(adj) | Training Dataset | | External Dataset | |
|---|---|---|---|---|---|
| | | RMSE | SRMSE | RMSE | SRMSE |
| Dataset | 0.64 | 32.45 | 0.40 | 39.49 | 0.61 |
| Dataset + imputation | 0.58 | 35.77 | 0.44 | 39.98 | 0.62 |

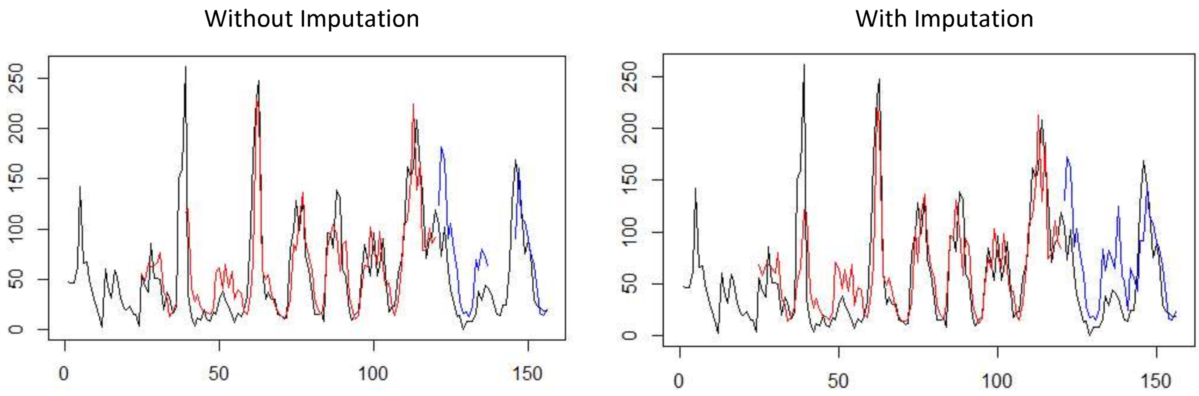


**S2 File_Fig 3.** Predicted Dengue Cases versus Reported Dengue Cases in 2001–2013
