# Supplementary material for: Prediction of Dengue Outbreaks Based on Disease Surveillance and Meteorological Data
Source: PLoS One. 2016 Mar 31;11(3):e0152688. doi: 10.1371/journal.pone.0152688 (PMC4816319; doi:10.1371/journal.pone.0152688)
Supplement: S3 File — (PDF) [file pone.0152688.s004.pdf]

## **S3 File.** Population Offset

Population data for study location is obtained from the Central Bureau of Statistics, Yogyakarta Province[1]. The annual population growth rate in Yogyakarta Municipality is below 1% during 1990-2010, thus the population was relatively stationary during the study period.

**S3 File_Table 1.** Population Growth Rate per Year in Yogyakarta Municipality

| Census | Pop | Growth Rate/year |
|---|---|---|
| 1961 | 306296 | *NA* |
| 1971 | 340491 | 0,0106 |
| 1980 | 398089 | 0,0174 |
| 1990 | 412059 | 0,0034 |
| 2000 | 396711 | -0,0038 |
| 2010 | 388627 | -0,0021 |

The Table below illustrate that adding population as offset in this case study does not change much due to the stable population in the study period.

**S3 File_Table 2.** Predictive Performance Statistics, without and with offset

| Offset contribution | R-sq.(adj) | AIC | Training Dataset | | External Dataset | |
|---|---|---|---|---|---|---|
| | | | RMSE | SRMSE | RMSE | SRMSE |
| Model D | 0.636 | 2311.60 | 32.448 | 0.403 | 39.491 | 0.613 |
| Model D + offset | 0.636 | 2315.32 | 32.476 | 0.404 | 38.966 | 0.605 |

[1] Badan Pusat Statistik (BPS) Provinsi Daerah Istimewa Yogyakarta. **Penduduk Provinsi Daerah Istimewa Yogyakarta Hasil Sensus Penduduk 1961-2010** [Internet]. Yogyakarta: Badan Pusat Statistik (BPS) Provinsi Daerah Istimewa Yogyakarta; 2013 [cited 2015 Dec 10]. Available from: http://yogyakarta.bps.go.id/website/pdf_publikasi/Penduduk-Provinsi-Daerah-Istimewa-Yogyakarta-Hasil-Sensus-Penduduk-1961-2010.pdf. Indonesian.
