# Supplementary material for: Prediction of Dengue Outbreaks Based on Disease Surveillance and Meteorological Data
Source: PLoS One. 2016 Mar 31;11(3):e0152688. doi: 10.1371/journal.pone.0152688 (PMC4816319; doi:10.1371/journal.pone.0152688)

S4 File. Comparison of Prediction Accuracy of Several Outbreak Criteria

For the final prediction model derived, we established predictions and calculated the accuracy of the prediction according to a binary classification of months as outbreaks or non-outbreaks. We compared prediction accuracy according to sensitivity, specificity, and positive and negative predictive values of the final prediction model when it was applied to the following outbreak threshold:

1. Constant outbreak threshold: the average of monthly dengue cases during study period (2001-2010), and the incidence rate (>300/100,000)
2. Moving outbreak threshold: the definition of a dengue outbreak in Indonesia[2] and by WHO[3]

S4 File_Table 1. The Comparison of the Prediction Accuracy

Prediction accuracy	constant outbreak threshold		moving outbreak threshold	
	monthly average cases 2001-2010 = 60 cases	IR > 300/100,000	2 * dengue lag12 (Badurdeen et al., 2013)	monthly average cases of the preceding 5 years + 2 SD (WHO, 2009)
Sensitivity	88.9 %	outbreak never existed, according to the surveillance	75.0 %	50.0 %
Specificity	81.0 %		84.3 %	98.2 %
PPV	74.4 %		62.1 %	66.7 %
NPV	92.2 %		90.8 %	96.5 %

S4 File_Fig 1. Outbreak Threshold, Observed and Predicted Dengue Cases from 2001–2010

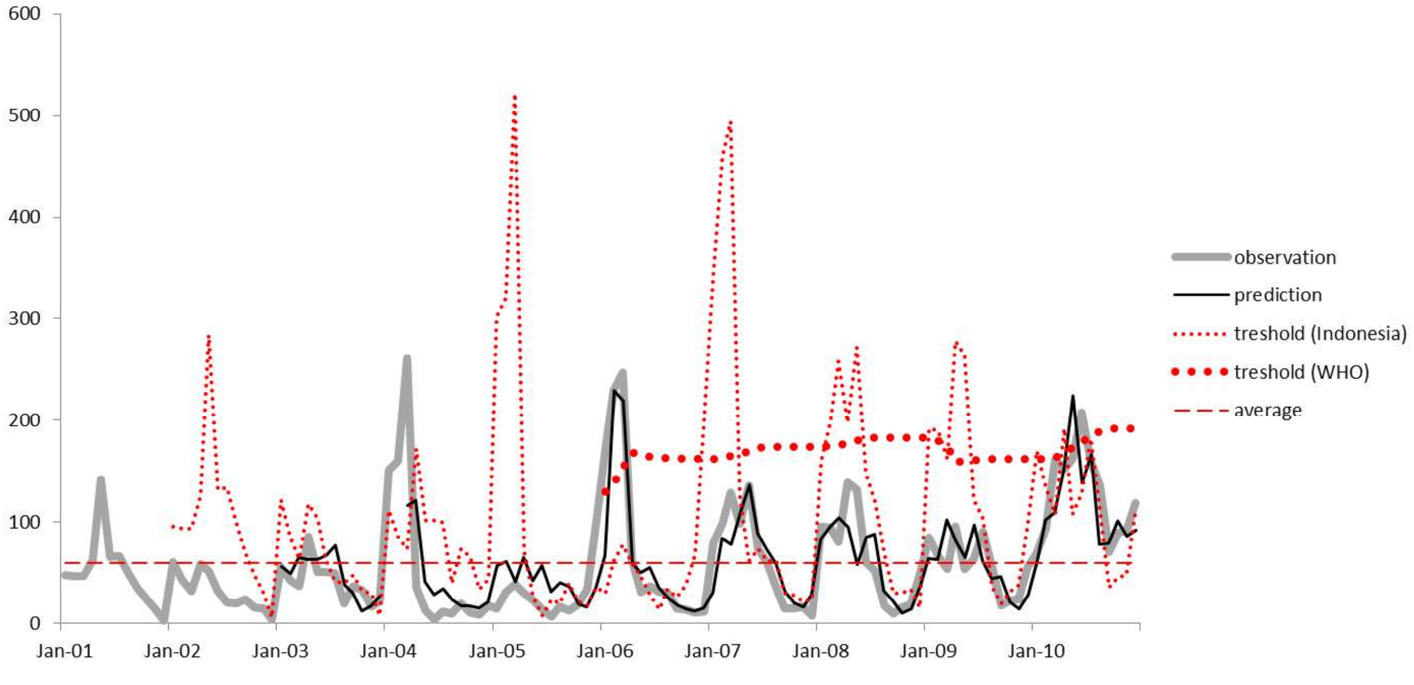

[2] Badurdeen S, Valladares DB, Farrar J, Gozzer E, Kroeger A, Kuswara N, et al. Sharing experiences: towards an evidence based model of dengue surveillance and outbreak response in Latin America and Asia. **BMC public health**. 2013;13(1):607

[3] World Health Organization. **Dengue: guideline for diagnosis, treatment, prevention and control**. Geneva: World Health Organization; 2009

S4 File_Fig 2. Outbreak Threshold (300 cases per 100.000) and Monthly Incidence (per 100.000) from 2001–2010

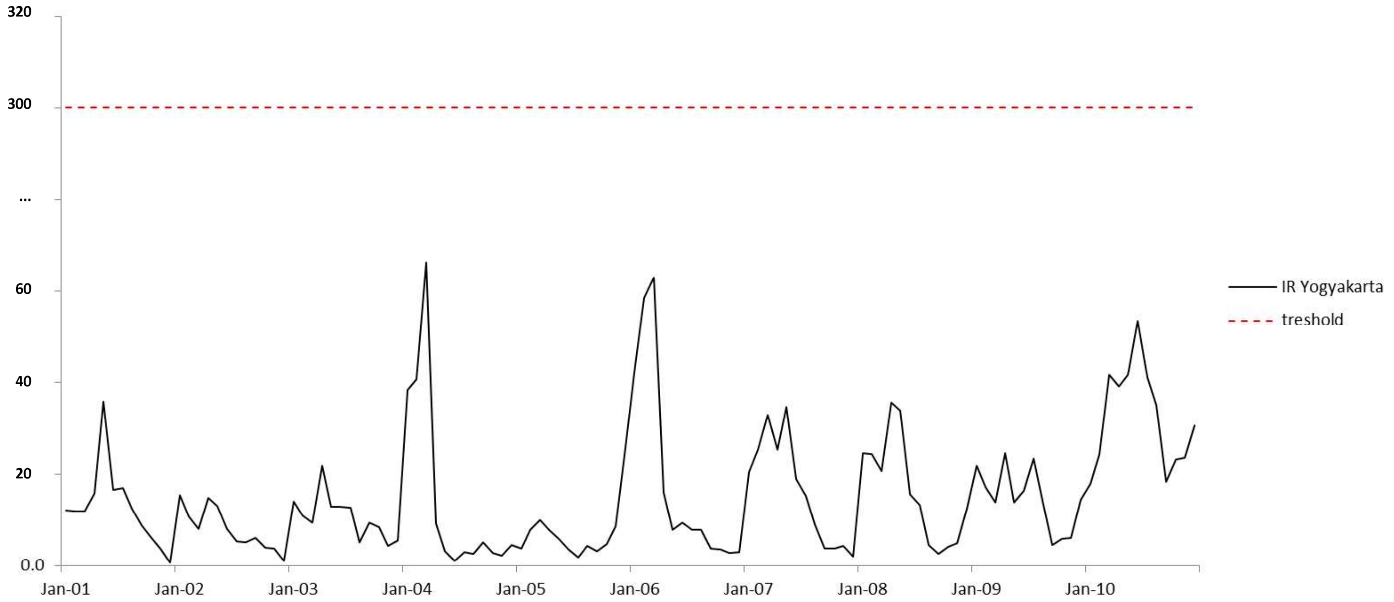

Supplement: S4 File — (PDF) [file pone.0152688.s005.pdf]
