# Supplementary material for: Prediction of Dengue Outbreaks Based on Disease Surveillance and Meteorological Data
Source: PLoS One. 2016 Mar 31;11(3):e0152688. doi: 10.1371/journal.pone.0152688 (PMC4816319; doi:10.1371/journal.pone.0152688)
Supplement: S1 Table — (PDF) [file pone.0152688.s006.pdf]

S1 Table. Initial Analyses Summary

Results				
**P-value**	**Lag0**	**Lag1**	**Lag2**	**Lag3**
- Temperature	0.8305	0.9730	0.5327	0.0326*
- Rainfall	0.2338	0.2149	0.0227*	0.0118*
- Adjusted Humidity	0.4802	0.4249	0.7811	0.3414
**R-sq.(adj)**	0.31			
**RMSE**	41.468			
**SRMSE**	0.516			
